# Effects of forest management on native bee biodiversity under the tallest trees in the world

**DOI:** 10.1002/ece3.10286

**Published:** 2023-07-09

**Authors:** Nya Ealy, Jaime Pawelek, Jenny Hazlehurst

**Affiliations:** ^1^ California State University East Bay Hayward California USA; ^2^ Essig Museum of Entomology, Valley Life Sciences Berkeley California USA

**Keywords:** biodiversity, Hymenoptera, interaction networks, redwood forest, restoration

## Abstract

It is not clear if mature secondary growth coniferous forests can support similar pollinator communities as old growth coniferous forests, or how active management (e.g., retention forestry) in mature secondary growth forests may affect pollinator communities. We compare the native bee community and plant‐bee interaction networks of old growth, naturally regenerating and actively managed (retention forestry) mature secondary growth forests of similar stand age. Old growth forests had a higher bee species richness and Shannon's diversity index, but not Simpson's diversity index, than both actively managed and naturally regenerating mature secondary forests. Forest type (old‐growth, naturally regenerating mature secondary growth, and actively managed mature secondary growth) had a significant effect on bee community composition. Redwood forest bee‐plant interaction networks were small in size and had lower complexity than expected and few connector species. While studies suggest that small‐scale timber harvest may increase bee biodiversity in the short‐term in other coniferous forest habitats, our study suggests that there may be long‐term negative effects of clear‐cutting that lower bee biodiversity in mature secondary growth forests as compared to mature old‐growth forests.

## Graphical abstract

We investigated native bee biodiversity in coast redwood forests in California. We found significant differences in bee community diversity and composition, and in plant‐pollinator interactions between old growth, secondary growth, and actively managed redwood forests.

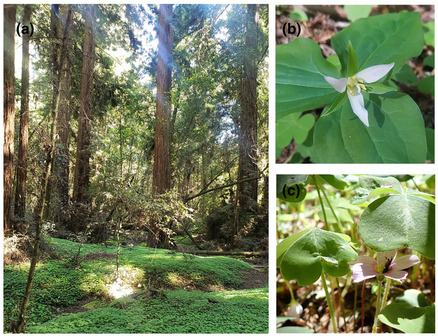

## 1 INTRODUCTION

Coast redwood (*Sequoia sempervirens*) forests are a unique ecosystem within the global biodiversity hotspot of the California Floristic Province (Myers et al., 2000) that include the tallest trees in the world and are associated with many unique flora and fauna. Redwood forests have undergone intensive historical clear‐cutting, and over the last 150 years 95% of mature primary (old growth) redwood forests were clear‐cut (Noss, 2000). The majority of redwood forests today are mature secondary growth forests that have regenerated from large clear‐cut areas. These secondary growth forests include naturally regenerating forests and areas that are actively managed through techniques like retention forestry, where for example, portions of tree stands are left un‐cut in order to retain structural and compositional forest biodiversity. It is not known if critical ecological processes, such as the pollination of understory flowering plants by bees, is comparable in mature secondary growth redwood forests to mature old‐growth redwood forests, or how common active management methods like retention forestry in mature secondary growth forests might affect native bees.

Historical clear‐cutting and retention forestry can have a wide range of effects on the redwood forest ecosystem, including soil compaction and erosion, changes in the amount of large woody debris and dead tree snags, and introduction of non‐native or invasive plant species into canopy gaps left behind after trees are harvested (Russell & Jones, 2001). A study on managed early successional coniferous forests in western Oregon that have had residue from timber harvest removed suggests that active management may increase the biodiversity of bees in terms of abundance and observed species richness (Rivers et al., 2018). Several studies on active management forestry practices in European forests suggest that the structural complexity of habitat, including canopy cover, stand structural complexity, and standing dead wood are all important indicators of cavity‐nesting bee and wasp diversity (Eckerter et al., 2021; Rappa et al., 2023). However, no such research has been done in coast redwood forests, and this information is crucial to effective restoration and management.

**FIGURE A1:**
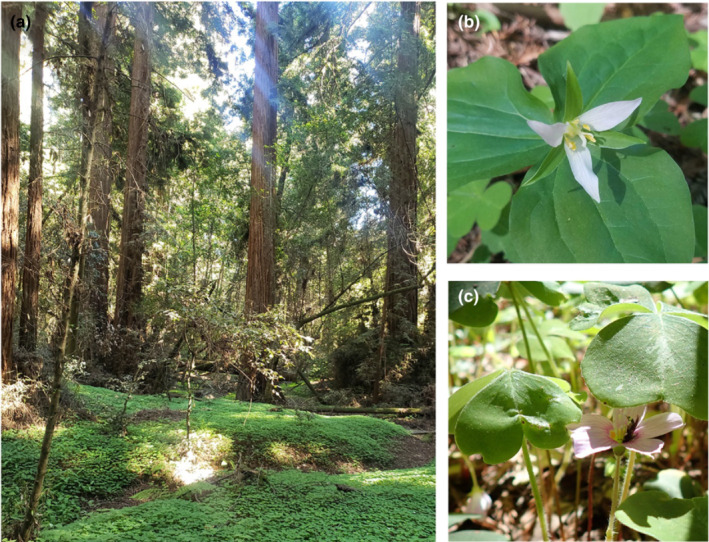
The old growth redwood forest at Henry Cowell State Park showing (a) A patch of redwood sorrel on the forest floor, (b) old growth redwood forest associated flowering plant *Trillium ovatum*, and (c) a species of native bee, possibly *Lasioglossum* sp., visiting redwood sorrel (*Oxalis oregana*) under leaf cover.

Old‐growth redwood forests are characterized by a diverse size and age distribution of redwood trees, gallery‐like open spaces between trees, and naturally occurring gaps in the canopy where older trees have fallen, leaving space for seedlings to grow (Figure A1: Appendix). Light levels in the understory range from 30% to 75% that of light levels above the canopy in dense redwood forests, and from 40% to 90% that of light levels above the canopy in dispersed redwood forests (Berrill et al., 2018). Climax flowering plants of the mature old‐growth coast redwood forest understory include the shrub *Rhododendron macrophyllum* and several shade‐adapted understory flowering plants (Figure A1: Appendix). Of these flowering plant species, some are known to be sensitive to timber harvest, including *Oxalis oregana* (redwood sorrel), *Trillium ovatum* (Pacific trillium), *Lysimachia latifolia* (Pacific starflower), and *Viola sempervirens* (redwood violet; Russell, 2009, 2020; Russell & Michels, 2011). Little detailed data on native bees is available from coast redwood forest ecosystems. However, Beattie (1971) described observations of small bees in the Andrenidae and Halictidae families, hoverflies (Family Syrphidae), and butterflies visiting redwood violets, and noted that pollinator activity followed the movement of sun‐spots along the forest floor. Gujral et al. (2022) documented native bees visiting Dudley's Lousewort (*Pedicularis dudleyi*), a rare and endangered redwood plant. Mature secondary growth redwood forests (101–130 years since clear‐cutting) are characterized by a lower age and size diversity of trees and higher tree density than in old growth forests, smaller large woody debris (LWD), and a lower diversity and coverage of native understory herbs and shrubs (Russell, 2020; Russell & Michels, 2011). Large woody debris from fallen mature trees or branches and deadwood can provide nesting substrate for cavity‐nesting bees in forests (Rappa et al., 2023). The higher stem density of secondary forests can result in deeper leaf litter (Willett, 2001), which in addition to higher tree density, allows for less exposed bare ground, which has a significant effect on the abundance and diversity of ground‐nesting bees in other habitats (Decker & Harmon‐Threatt, 2019; Quistberg et al., 2016). Naturally regenerating mature secondary growth forests may thus have a lower abundance, species richness, Shannon's diversity, and Simpson's diversity index of native bee species than old‐growth forests due to lower availability of flowering plants and nesting substrate.

Active management of secondary growth redwood forests through retention forestry can cause some harvest effects on forest structure, such as increased soil compaction and the introduction of non‐native plant species (Russell, 2020). A study by Hanover and Russell (2018) found that tree canopy cover and native understory flowering plant species cover and richness were significantly higher in naturally regenerating as compared to actively managed redwood stands. A study by Petersen and Russell (2017) found that retention forestry in mature secondary growth redwood forests at levels of 22.78% or higher of stand harvested led to significant reductions in mature old‐growth redwood forest characteristics such as the density of LWD, native understory plant species abundance, and canopy cover. Actively managed (AM) redwood forests may thus have a distinct bee community and plant‐pollinator interaction structure from naturally regenerating (NR) secondary growth forests and old growth (OG) forests due to reductions in native flowering plant availability and nesting substrate. Active management methods vary in forestry, but in other systems they have had a significant effect on the forest structure, bee communities, and nesting substrate availability for bees. Rodríguez and Kouki (2015) found that bee community composition in managed boreal forests in Europe was determined by percentage of bare ground and number of logs in the area, and that forest management through prescribed fire increased the availability of these habitat types. In a subsequent study, they determined that intermediate levels of management‐related disturbance, including prescribed fire and timber harvest, positively increased the invertebrate pollinator diversity of actively managed forests (Rodríguez & Kouki, 2015). In Japanese cedar forests, selective harvest led to an initial increase in pollinator biodiversity, however several years later the communities in AM forests were no longer distinct from NR forests (Taki et al., 2010).

In this study, we compare native bee species richness, Shannon's diversity index, Simpson's diversity index, and community composition in AM secondary growth, NR secondary growth, and OG redwood forests of the Santa Cruz mountains in California. We predicted that native bee biodiversity would be lower in AM and NR forests than in OG forests due to decreases in floral resource and nesting substrate availability for both cavity‐ and ground‐nesting bees, and that bee community composition would be significantly different between forest types.

We also analyzed bipartite plant‐native bee interaction networks for each type of forest. Interactions between pollinators that specialize on very few plant species, or vice versa, are generally more likely to be lost due to landscape changes like deforestation (e.g., Ferreira et al., 2020), which can change network structure and resilience to future anthropogenic disturbance. We predicted that pollination networks in old growth forests would exhibit higher nestedness, connectance, specialization, and robustness than randomly generated null model networks of the same size, while networks from actively managed and secondary growth forests would exhibit lower nestedness, connectance, specialization, and robustness than null models. We also identify potential plant and pollinator species of interest for targeted conservation efforts based on their centrality within interaction networks.

## 2 MATERIALS AND METHODS

### 2.1 Study location

**FIGURE 1:**
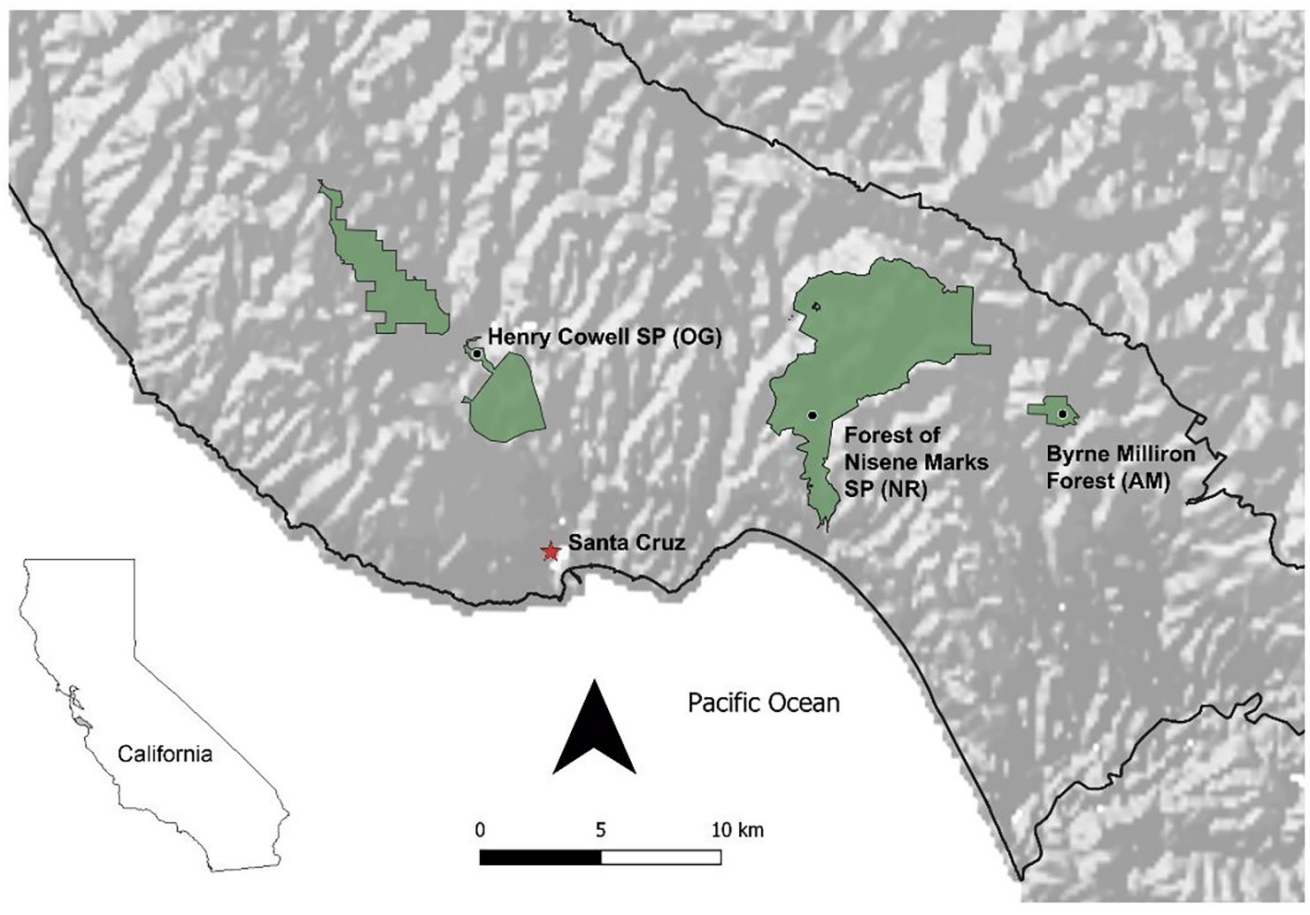
Map of study sites in Santa Cruz Mountains of California on the west coast of N. America. Points indicate general area of study, however extent of study areas are larger than a single point. The city of Santa Cruz and location of Santa Cruz County within the state of California are shown for reference. AM, actively managed secondary forest; NR, naturally regenerating secondary forest; OG, remnant old growth forest.

This study took place in the Santa Cruz mountains of California in OG redwood forest, NR mature secondary growth forest, and actively managed through retention forestry (AM) mature secondary growth forest of similar stand age (~120 years old; Figure 1). We conducted three sampling trips per year to each forest type between March and May of 2020–2021 to encompass the peak bloom period for understory flowering plants in this ecosystem. We sampled OG forest in Henry Cowell State Park, a 1881‐ha park dominated by naturally regenerating secondary growth forest, but which does contain a remnant patch of old growth forest. This is the only remaining substantial old growth forest in the Santa Cruz mountains, as the nearby Big Basin State Park experienced a severe fire in 2020 during the CZU lightning complex fires. We sampled NR forest in the Forest of Nisene Marks State Park, a 4046‐ha park which has an average stand age of approximately 120 years. We sampled AM forest in the 66‐ha Byrne‐Milliron Forest, which is managed by the Land Trust of Santa Cruz County. The Byrne‐Milliron Forest has an average stand age of approximately 130 years, and has been managed with techniques including retention forestry since the 1980s, with the most recent harvest in the area sampled occurring in 2007.

### 2.2 Bee sampling plots

In order to capture bees during foraging, we first located patches of flowers in the redwood forest understory. Patches of flowers were often small and dispersed from one another. Field crews searched for flowers in the forest in a zig‐zag pattern until patches of flowers with at least one active bloom were found. Once a patch of flowers was located, a circular bee sampling plot with a diameter of 10 m was placed at that location with its center over the highest density of blooms in the flower patch. All plots were at least 15 m from trails or roads, and were at least 15 m apart.

### 2.3 Environmental data

The following environmental data were collected at each bee sampling plot: the number of redwood trees per size class of diameter at breast height (DBH) for all trees >7 cm within a 20 m diameter circle from the center point of the bee sampling plot, number of LWD defined as any individual pieces of dead wood >10 cm in diameter at the widest point within a 20 m diameter circle, and canopy cover at the plot center measured with a spherical densiometer. We counted the number of trees within estimated DBH size classes of >152, 127–152, 101–126, 76–100, 50–75, 25–49, and 7–24 cm, and used these values to calculate a minimum basal area of redwood trees in each plot using the standard equation (basal area = DBH^2^ × 0.005454). To quantify the floral resources available, we counted the number of blooms of each plant species present within the plot at the start of sampling. We sampled plots in each forest type three times, in March, April, and May of 2021–2022, with approximately 3 weeks in between sampling periods. As the distribution of active blooms shifted over the course of our sampling period, new plots were located and sampled at each visit depending on where flowers were blooming.

### 2.4 Timed bee sampling in plots

All sweep‐net sampling occurred between 11:45 am and 5:00 pm, which was when we observed peak bee activity at blooms. Sampling only occurred when temperatures were above 18°C, and wind speeds were below 3.6 m/s. Plots were sampled continuously for 30 min by a single person with a sweep net, and any bee that landed on any flower part (petals, anthers, stigma, petioles) of any bloom within the bee sampling plot was captured. Introduced European honey bees (*Apis mellifera*) were observed in low numbers at all forest types, but were not included in this study and were not captured. Specimens were placed in kill jars containing ethyl acetate, pinned, labeled with the date, plot ID, forest type, geographic coordinates, the initials of the collector, and the plant species on which it was captured. Specimens were sent to a taxonomic expert for identification to the lowest taxonomic level possible (Table A1: Appendix). There are many bee taxa that lack species‐level identification keys in western N. America. If it was not possible to key a specimen out to the species level, then specimens were grouped into morphospecies based upon key morphological similarities (Table A2: Appendix). We also attempted passive sampling of bees with “bee bowl” or “pan trap” stations consisting of yellow, blue, and white bowls half‐filled with soapy water (Shapiro et al., 2014) and distributed randomly on the forest floor, however bee specimen yield was too low to warrant analysis (Table A3: Appendix), and this does not appear to be a useful method for sampling bees in redwood forests.

### 2.5 Environmental analysis

To identify important differences between forest types in terms of environmental variables that could affect bee biodiversity, we used Kruskal–Wallis tests in the statistical program R (R Core Team, 2021) to detect significant differences between forest types in terms of the following variables: canopy cover, minimum basal area of redwoods, the number of redwood trees in the plot with DBH > 152 cm, the number of LWD, the number of blooms of the most common redwood understory flower, redwood sorrel, and the total number of open blooms of all plant species in each plot.

### 2.6 Bee biodiversity analysis

To determine which bees were most common in each forest type, we calculated the average number of specimens of each bee species captured per plot and ranked them from most to least frequently captured. We used the number of bees captured per plot to calculate coverage‐based extrapolation curves for each forest type with the function “iNEXT” in the package “iNEXT” at a 95% coverage level (Chao & Jost, 2012). We used “iNEXT” to calculate the species richness (Hill number *q* = 0), Shannon diversity (Hill number *q* = 1), and Simpson diversity (Hill number *q* = 2; Chao & Jost, 2012). The Hill number and coverage‐based estimation approach is now commonly used in biodiversity analyses due to its numerous benefits over traditional biodiversity index analyses, including resilience to unbalanced sampling and small sample sizes (Roswell et al., 2021). Species richness gives equal weight to rare species and common species, Shannon's diversity index gives more weight to common species, and Simpson's diversity index is interpreted as the number of very abundant species only. To understand the differences in bee community composition between forest types, we used the metaMDS function of the R‐package “vegan” (with 1000 permutations; Oksanen et al., 2020). We used non‐metric dimensional scaling (NMDS) of a Bray–Curtis dissimilarity matrix to visualize any differences in species composition. We then used PERMANOVA (Permutational multivariate analysis of variance) in the adonis function of the “vegan” package (Oksanen et al., 2020) and calculated pairwise differences using the pairwise. adonis function with Bonferroni correction in the “pairwise. adonis” package (Martinez, 2020). We used PERMDISP (Permutational analysis of multivariate dispersions) to test for homogeneity of variances using the function betadisper in the package “vegan.”

### 2.7 Plant‐bee interaction network analysis

Networks per plot were too small to calculate network indices, so cumulative weighted bipartite networks for each forest type were generated based on the mean number of each bee species captured on each flowering plant species across all plots in a forest type. We calculated the following network‐level indices for each forest type using the function networklevel in the package “bipartite” (Dormann et al., 2009): connectance, nestedness, specialization, and robustness. Nestedness quantifies the extent to which specialist pollinators interact with a subset of the network that includes more generalist pollinators (Bascompte et al., 2003). Connectance measures the proportion of all possible interactions that are observed. Specialization measures the non‐random preference of plants and pollinators for one another across the network. Robustness tests how resilient a network is to randomized removal of species from the network (Blüthgen, 2010). We measured the following species‐level network indices: degree (the number of other species that a species interacts with), closeness centrality (the inverse of the sum of the distance between the species of interest and every species in the network), and betweenness centrality (a measure of how often the species of interest lies on the shortest path between any other two species in the network; Blüthgen, 2010). Network indices are sensitive to network size, and so empirical network indices were compared to randomly generated null model networks (e.g., Blüthgen et al., 2008; Burkle & Knight, 2012; Dormann et al., 2009; MacLeod et al., 2016). Null model networks of the same size as each empirical network were randomly generated 1000 times using two different available methods of null model generation (r2d and shuffleweb) using the function “nullmodel” in the package “bipartite” (Dormann et al., 2009). We calculated *z*‐scores comparing empirical and null model values for each network index and calculated *p*‐values to determine if network indices differed significantly from null models. Network *z*‐scores were not directly compared between forest types because plot‐level networks were too small to calculate indices (likely due to low species diversity and dispersed nature of flowering patches in the understory) and thus cumulative forest type networks were analyzed instead, giving us a sample size too low (*N* = 1 network per forest type) for statistical analysis. We also calculated the following species‐level indices of network centrality for plants and bees using the function specieslevel in the package “bipartite” (Dormann, 2011) to identify species of importance to network stability: normalized degree, weighted betweenness centrality, and weighted closeness centrality.

## 3 RESULTS

Between 2021 and 2022 we sampled 60 plots in the understory of AM mature secondary growth forest, 74 plots in NR mature secondary growth forest, and 82 plots in mature OG redwood forests in the Santa Cruz mountains. Sample sizes reflect the lower abundance of blooms in AM and NR forests as compared to OG forests, and equal effort (180 person hours per forest type) was taken searching for flowers in all forest types.

### 3.1 Environmental analysis

Forest types showed significant variation in environmental variables (Table 1). Canopy cover was significantly different between forest types, and was highest in the NR forest (Kruskall–Wallis chi‐squared = 6.95, *p* < .05). The estimated minimum basal area of redwood trees per plot was significantly greater in OG forest, followed by AM and NR (Kruskall–Wallis chi‐squared = 60.32, *p* < .001). The number of redwood trees per plot with DBH > 152 cm was significantly greater in OG forest, followed by AM and NR forests (Kruskall–Wallis chi‐squared = 91.42, *p* < .001). The number of LWD per plot was not significantly different between forest types (Kruskall–Wallis chi‐squared = 5.75, *p* > .05). The number of redwood sorrel blooms per plot was significantly greater in OG forest, followed by NR and AM forests (Kruskall–Wallis chi‐squared = 51.32, *p* < .001). The total number of flowers per plot was significantly greater in OG forest, followed by NR and AM forests (Kruskall–Wallis chi‐squared = 53.83, *p* < .001).

**TABLE 1:** Comparison of environmental variables in bee sampling plots from old‐growth (OG), naturally regenerating secondary growth forest (NR), and actively managed secondary growth forest (AM).

Environmental variable	Forest type		
	AM (*N* = 60)	NR (*N* = 74)	OG (*N* = 82)
Canopy cover*	70.62 (22.08)	80.77 (13.96)	76.97 (15.42)
Minimum basal area of Redwoods***	34.02 (21.42)	21.03 (14.85)	62.02 (41.18)
Num. of Redwoods DBH > 60 per plot***	0.69 (0.93)	0.21 (0.47)	2.52 (2.06)
LWD*	3.33 (3.31)	5.00 (4.48)	3.81 (4.45)
Num. of O. oregana blooms***	9.43 (11.73)	27.51 (40.31)	69.67 (60.14)
Total num. of flowers***	15.70 (15.11)	37.72 (41.09)	78.23 (63.90)

*Note*: Values shown are mean (SD). Sample size (number of plots, *N*) are shown next to each forest type to indicate the number of bee sampling plots sampled in each forest type. Environmental variables that varied significantly between forest types based on a Kruskal–Wallis test are indicated in asterix, where * indicates *p* < .05 and *** indicates *p* < .001.

### 3.2 Native bees identified

**FIGURE A2:**
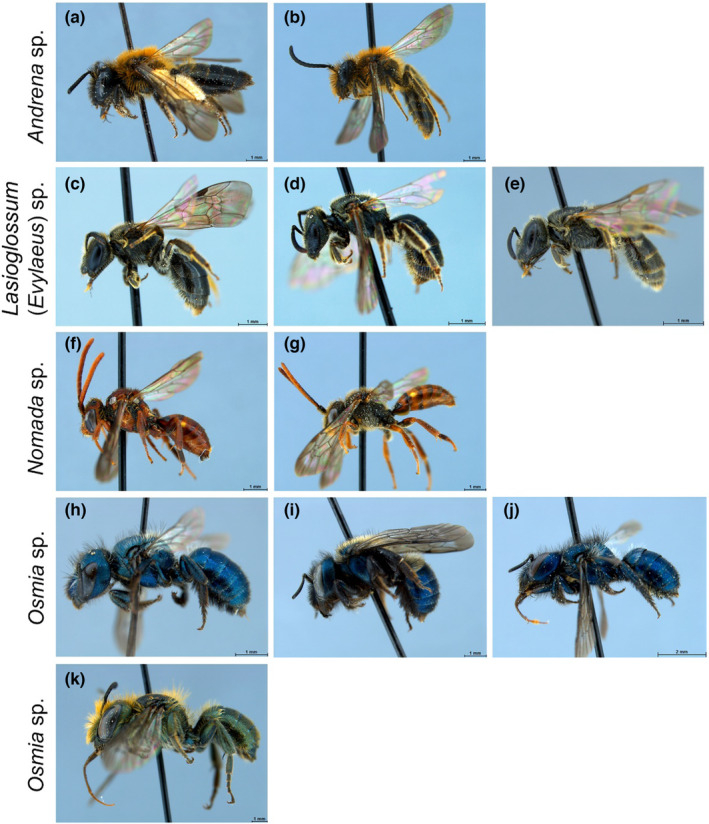
Genus‐level bee species found in redwood forests (a) *Andrena* sp. female, (b) *Andrena* sp. male, (c) *Lasioglossum* (*Evylaeus*) sp. A female, (d) *Lasioglossum* (*Evylaeus*) sp. B female, (e) *Lasioglossum* (*Evylaeus*) sp. E female, (f) *Nomada* sp. female, (g) *Nomada* sp. male, (h) *Osmia* (*Melanosmia*) sp. A female, (i) *Osmia* (*Melanosmia*) sp. B female, (j) *Osmia* (*Melanosmia*) sp. C female, (k) *Osmia* sp. male. Background is Pantone Neutral Gray in all specimen photos, and scale bars are in mm.

We captured a total of 106 bees across all forest types and bee sampling plots over 2 years of sampling. These bees included specimens from the Apidae, Halictidae, Andrenidae, and Megachilidae families. We identified a total of 12 distinct species of bees across all three forest types (Table A1: Appendix), 4 of which were identified to the species level, and 8 to genus (Figure A2: Appendix), within which there were six distinct morphospecies (Figure A2; Table A2: Appendix). Native bees in the redwood forest understory were illusive and difficult to find, and we observed them frequently hiding underneath the leaves of redwood sorrel until a sun beam hit their area, at which point they began to forage actively (Figure A1: Appendix). Each forest type showed different patterns of abundance in the most frequently captured bee species (number of individuals/plot), with *Andrena* sp. (Family Andrenidae) and *Nomada* sp. (Family Apidae) as the only species that occurred across all three forest types (Table 2). The *Andrena* species could not be identified based on any available species‐level keys (J. Pawelek, personal communication) and warrants further taxonomic investigation given the unique nature and limited area of the redwood forest ecosystem. Bees in the genus *Nomada* are kleptoparasites, and no key exists for them in western North America. *Nomada* species are known to parasitize many *Andrena* species (Stephen et al., 1969). We also found several *Osmia* (*Melanosmia*) and *Lasioglossum* (*Evylaeus*) species that could not be keyed out to the species level. Given the uniqueness of the coastal redwood forest ecosystem, we recommend further study to determine the taxonomic status of these species. We found both ground‐ and cavity‐nesting bees. Most *Lasioglossum* and *Andrena* are ground‐nesting. *Ceratina acantha* is a cavity‐nesting species, as are bees of the genus *Osmia*, using pithy stems and even rocks or snail shells as nesting substrate.

**TABLE 2:** Relative frequency of capture of bee species in each forest type.

Forest type	Species	Mean (SD)
AM (*N* = 65 plots)	*Nomada* sp.	0.11 (0.36)
	*Andrena* sp.	0.03 (0.18)
	*Bombus vosnesenskii*	0.02 (0.13)
NR (*N* = 75 plots)	*Andrena* sp.	0.13 (0.38)
	*Nomada* sp.	0.12 (0.57)
	*Lasioglossum* sp. A	0.07 (0.30)
	*Osmia* sp.	0.05 (0.28)
	*Lasioglossum* sp. B	0.01 (0.12)
OG (*N* = 83 plots)	*Lasioglossum* sp. A	0.29 (0.62)
	*Andrena* sp.	0.18 (0.50)
	*Nomada* sp.	0.09 (0.48)
	*Bombus melanopygus*	0.07 (0.31)
	*Ceratina acantha*	0.07 (0.47)
	*Osmia* sp.	0.05 (0.22)
	*Osmia* sp. A	0.02 (0.16)
	*Halictus tripartitus*	0.01 (0.11)
	*Lasioglossum* sp. E	0.01 (0.11)
	*Osmia* sp. C	0.01 (0.11)

*Note*: Values shown are mean numbers of native bees species captured per plot with standard deviations in parentheses (SD) in each forest type. Number of plots (sample size *N*) sampled in each forest type are shown in parentheses.

Abbreviations: AM, actively managed secondary growth forest; NR, naturally regenerating secondary growth forest; OG, old growth forest.

### 3.3 Bee biodiversity

**FIGURE 2:**
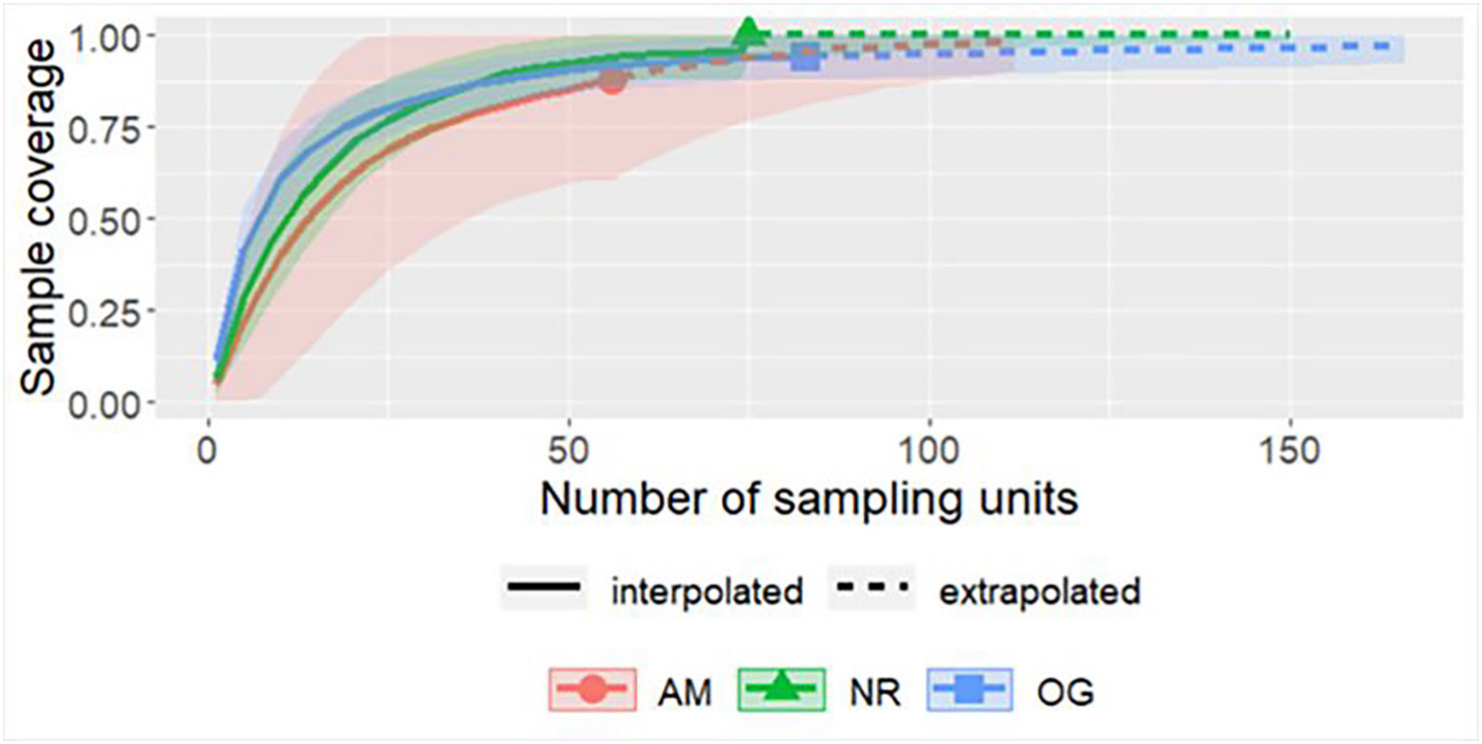
Species accumulation curves at a coverage‐based estimation level of 0.95. AM, actively managed secondary forest; NR, naturally regenerating secondary forest; OG, remnant old growth forest.

**FIGURE 3:**
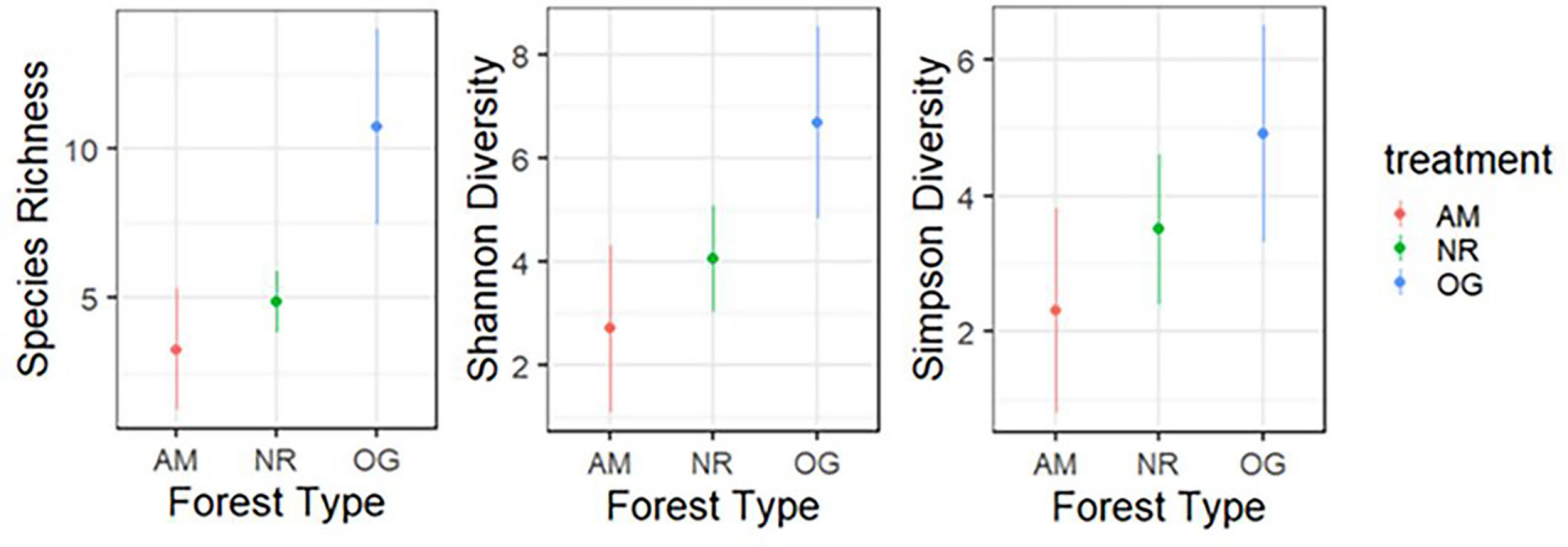
Species richness, Shannon diversity, and Simpson diversity values at a coverage of 95% across forest types, with 95% CI. AM, actively managed secondary forest; NR, naturally regenerating secondary forest; OG, remnant old growth forest.

**FIGURE 4:**
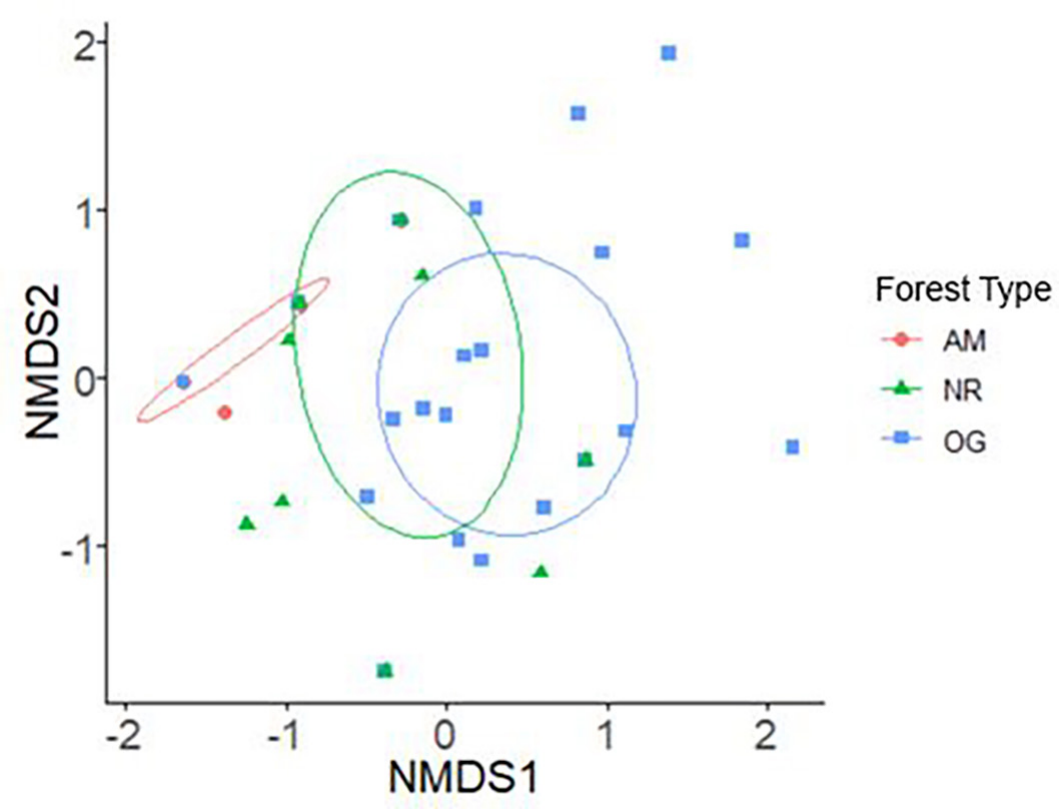
NMDS plot showing community composition of native bees by forest type; ellipses represent 95% CI. AM, actively managed secondary forest; NR, naturally regenerating secondary forest; OG, remnant old growth forest.

Abundance‐based accumulation curves flattened off for all three forest types (Figure 2). Old growth forest had non‐overlapping 95% confidence intervals for species richness higher than both AM and NR forests, but there were no other non‐overlapping pairwise comparisons across all diversity indices (Figure 3; Table A4: Appendix). The composition of bee communities differed significantly across forest types (PERMANOVA: *R* ^2^ = .14; *p* < .01; df = 2; Figure 4). Post‐hoc pairwise comparisons showed that bee communities in OG forest were significantly different from AM forests (*R* ^2^ = .16; *F* = 6.47; *p* < .01; df = 1). Forest type group dispersion was homogenous (PERMDISP: *F* = 2.07; *p* = .14; df = 0.14).

### 3.4 Plant‐bee interaction network analysis

**FIGURE 5:**
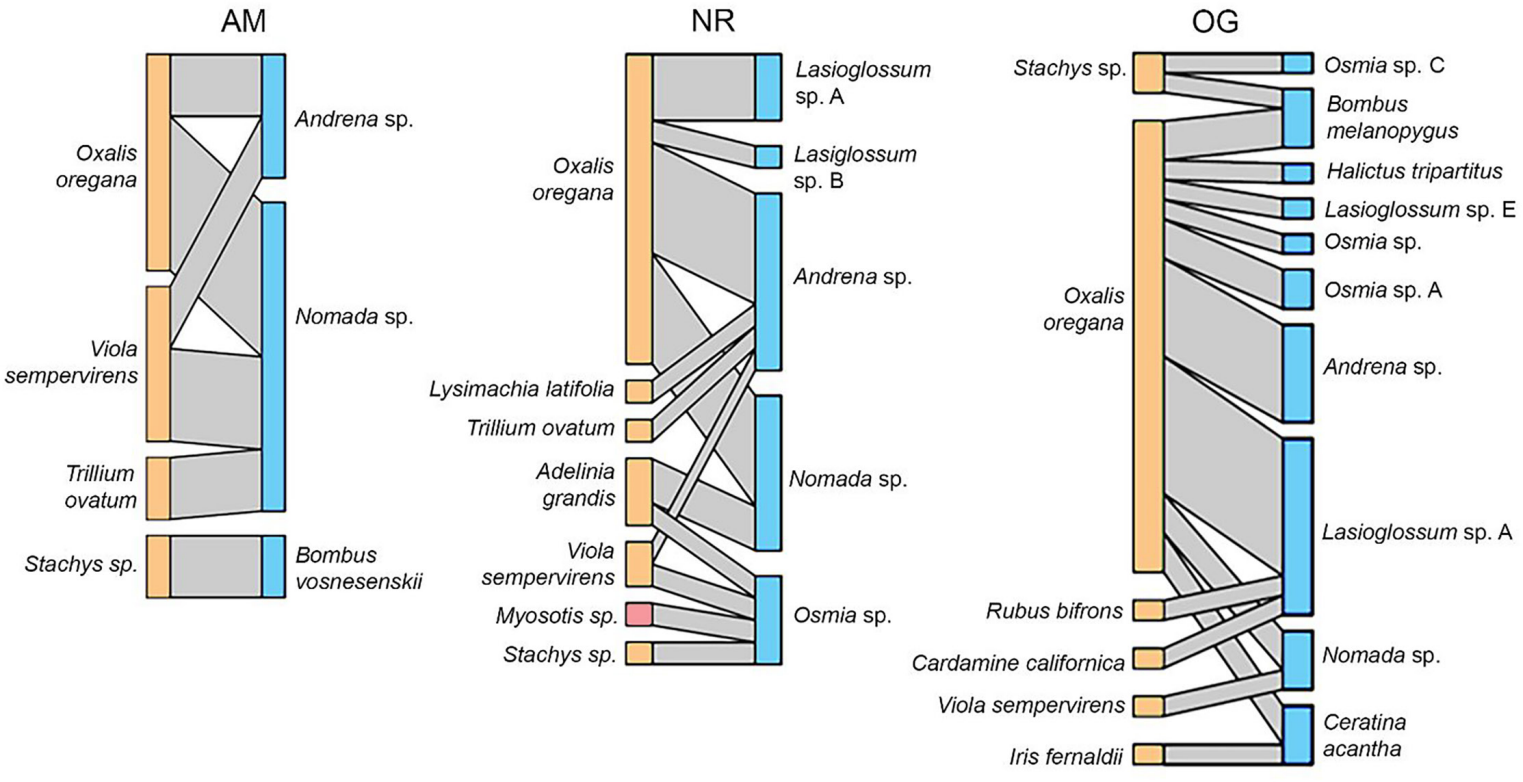
Interaction networks weighted by the mean number of specimens captured per plant per plot across forest types. AM, actively managed secondary forest; NR, naturally regenerating secondary forest; OG, remnant old growth forest. Plants are shown in orange on left, and native bee visitors in blue on right. Non‐native plant species are shown in red.

We calculated the nestedness, connectance, specialization (H2′), and robustness of weighted bipartite bee‐plant interaction networks (Figure 5; Table 3) and compared them to network indices for null models (1000 iterations) generated using the “r2d” and “shuffleweb” methods and calculating *z*‐scores (Table 4) to determine if network indices were significantly lower or higher than expected for a random network of the same size. Network *z*‐scores were not directly compared between forest types because plot‐level networks were too small to calculate indices and cumulative forest type networks were analyzed instead, giving us a sample size too low for statistical analysis. There was variation in *z*‐scores depending on which null model generation method was used. When the shuffleweb method was used to generate the null model, both NR and OG forest had lower than expected nestedness (z‐score = −2.18; *p* < .05; *z*‐score = −3.01; *p* < .001 respectively; Table 4). We found that using the r2d null model, AM forest had significantly lower connectance than expected (*z*‐score = −2.56; *p* < .05; Table 4). We found that using the r2d null model, AM forest had higher specialization than expected (*z*‐score = 2.03; *p* < .05; Table 4), while the shuffleweb null model method revealed that NR and OG forests both had significantly lower specialization than expected (*z*‐score = −3.57; *p* < .001; *z*‐score = −8.56; *p* < .001; Table 4). We found that using the shuffleweb null model, OG forest had significantly lower robustness than expected (*z*‐score = −5.08; *p* < .001; Table 4), but this pattern did not persist in the r2d null model analysis (Table 4).

**TABLE 3:** Network‐level plant‐pollinator interaction network indices (nestedness, connectance, specialization, and robustness) for interaction networks in different forest types.

Metric	Forest type		
	AM	NR	OG
Nestedness	36.04	22.08	21.91
Connectance	0.50	0.34	0.25
Specialization	0.52	0.31	0.19
Robustness	0.56	0.71	0.81

*Note*: Networks are weighted by the mean number of specimens of each species of pollinator captured per plot in each forest type.

Abbreviations: AM, actively managed secondary growth forest; NR, naturally regenerating secondary growth forest; OG, old growth forest.

**TABLE 4:** Results of a null model analysis of plant‐pollinator interaction network indices for each forest type.

Network metric	Null model method	Forest type		
		AM	NR	OG
Nestedness	r2d	1.24	−0.11	−0.07
	Shuffle	0.21	−2.18*	−3.01**
Connectance	r2d	−2.56*	−0.82	0.04
	Shuffle	na	na	na
Specialization	r2d	2.03*	0.95	−0.05
	Shuffle	−0.6	−3.57***	−8.56***
Robustness	r2d	−1.42	−0.12	1.28
	Shuffle	0.36	−1.43	−5.08***

*Note*: Values shown are *z*‐scores generated by comparing network indices calculated from the plant‐pollinator interaction network from each forest weighted by mean number of specimens of each species of pollinator captured per plot to the average metric calculated from 1000 iterations of null model networks of the same size (same number of plants and pollinators). *Z*‐scores for two different methods of null model generation (r2d and shuffle) are shown for each comparison. Significant z‐scores are shown in asterix, where * indicates *p* < .05, ** indicates *p* < .01, and *** indicates *p* < .001.

Abbreviations: AM, actively managed secondary growth forest; NR, naturally regenerating secondary growth forest; OG, old growth forest.

**FIGURE 6:**
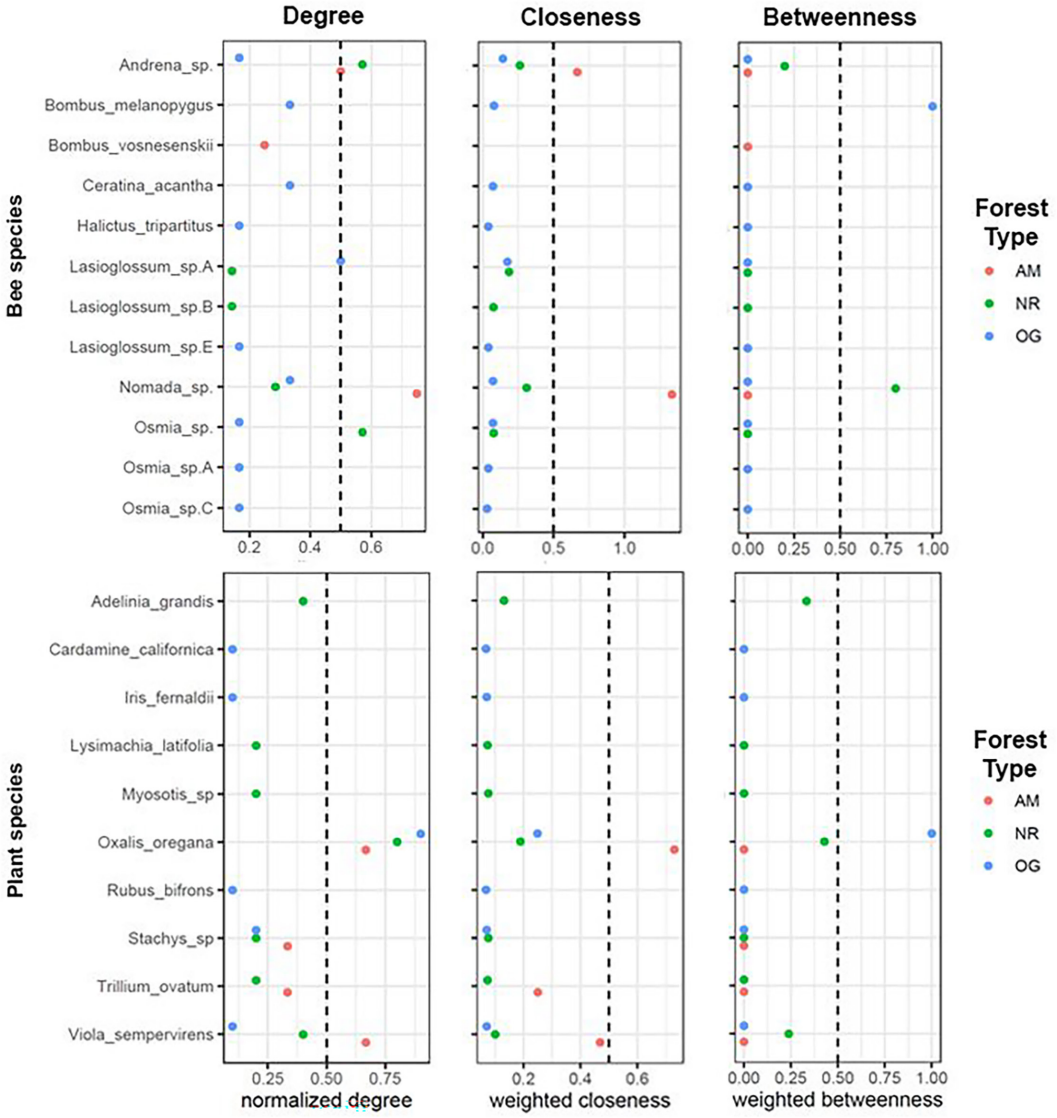
Centrality indices (normalized degree, closeness centrality, and betweenness centrality) based on cumulative interaction networks for each forest type. Forest types are indicated as AM, actively managed secondary forest; NR, naturally regenerating secondary forest; OG, remnant old growth forest. The dashed line at 0.5 is shown as a common reference point for comparison between indices.

Different plants and pollinators exhibited unique positions in their pollination networks at different forest types (Figure 6). Redwood sorrel had the highest normalized degree in OG and NR forests, while all other plants fell below 0.5. While redwood sorrel still had a normalized degree >0.5 in AM forests, it was surpassed by redwood violets. In contrast, in OG and NR forests redwood violets ranked <0.5 in normalized degree. In terms of weighted closeness, redwood sorrel ranked higher than all other plants in all forest types, though substantially more so in AM forests. In OG forests, all plants except for redwood sorrel had similar closeness values. All plants with a betweenness score of >0 serve as “connectors” within their networks, as they connect different interacting subsets of the network (González et al., 2010). Several plants emerge as connectors: redwood sorrel (OG and NR forests), *Adelinia grandis* (NR forest), and redwood violet (NR forest). AM forests had no connectors, likely due to the small size of that network.

For bee species, *Nomada* parasitic bees ranked highest in normalized degree in AM forests, but fell below 0.5 in NR and OG forests. In OG forests, *Lasioglossum* sp. A ranked the highest in normalized degree, though it ranked very low in NR forests and did not occur at all in AM forests. In NR forests *Osmia* ranked highest in normalized degree. In AM forests, *Nomada* bees and their likely hosts, *Andrena* bees, ranked highest in weighted closeness. In NR and OG forests, most species had similarly low closeness scores, however *Nomada* and *Andrena* ranked higher in NR forests than other species did. In OG forests, *Lasioglossum* sp. A ranked slightly higher than other species. Pollinators with a betweenness of >0 and are “connectors” were: *Bombus melanopygus* (OG forest), *Nomada* sp. (NR forest), and *Andrena* sp. (NR forest; Figure 6).

## 4 DISCUSSION

The bee fauna of mature redwood forests appears to be of relatively low abundance and diversity (Table A1: Appendix). As we predicted, old‐growth forests had a higher biodiversity of bee species than both naturally regenerating and actively managed mature secondary‐growth forest types in terms of species richness (Figure 3). Old‐growth forests also had higher Shannon's index than AM forests, but not NR forests, and not for Simpson's indices (Figure 3). This suggests that there are more rare species in OG forest as compared to in NR or AM forests, but that there are similar abundances of common species (e.g., *Andrena* and its parasite *Nomada*). While these conclusions are somewhat limited by our relatively small sample sizes, coverage‐based biodiversity estimation is resilient to small and/or unequal sample sizes (Roswell et al., 2021), and it is nonetheless important to gain a baseline understanding of native bee biodiversity in this important ecosystem. Bee biodiversity in secondary growth forests tends to decline with time since timber harvest as forests mature (Rivers & Betts, 2021; Rubene et al., 2015; Taki et al., 2018). This is likely due to the opening up of the canopy in early seral stage secondary growth forest that makes sunlight more available and results in the growth of a greater diversity of flowering plants, which in turn increase the biodiversity of bees. Some studies have suggested that bee pollination generally transitions to fly pollination as canopy cover increases (McCabe et al., 2019). Our data concur with this, suggesting that in both late seral stage secondary growth redwood forest and mature old growth forest bee biodiversity and abundance is relatively low. It is interesting that old growth forest had higher biodiversity than mature secondary growth forests, because this suggests that while early seral stage secondary‐growth redwood forests may have had higher bee biodiversity at some point in the past per the literature (Rivers & Betts, 2021; Rubene et al., 2015; Taki et al., 2018), there may be long‐term negative consequences for bee communities in late seral stage (mature) secondary growth forests following historic clear‐cutting. It is also of note that AM mature secondary growth forests showed a trend of lower bee biodiversity than NR mature secondary growth forests, as based on the literature retention forestry and active management should increase bee biodiversity over the short‐term following harvest (Pengelly & Cartar, 2010; Rivers & Betts, 2021). It is possible that the 14 year time interval since the last retention forestry harvest at our AM site was sufficiently long for any spike in bee biodiversity following harvest to have elapsed. In intensively managed douglas fir coniferous forests, bee diversity peaked 6–10 years post‐harvest and was lowest after 11 years post‐harvest once the canopy closed (Zitomer et al., 2023). However, the effects of active management on bees is dependent on the ecosystem and type of management, for example salvage logging has been shown to reduce bee abundance but not biodiversity (Galbraith et al., 2019).

As predicted, we also found significantly different species composition between forest types (Figure 4). These differences were driven by the higher variety of rare native bee species in OG forest, including several morphospecies of *Lasioglossum* (*Evylaeus*) and *Osmia* (*Melanosmia*), and *Ceratina acantha*, *Halictus tripartitus*, and *Bombus melanopygus*. This result contrasts somewhat with findings from secondary growth forests at different seral stages post‐harvest, in which changes in species composition were due to loss of species as the secondary growth forest matured rather than species turnover or species gain as in our system (Zitomer et al., 2023). It is possible that OG redwood forests are able to support unique bee species that are specialists in mature old‐growth forest habitats, but that long‐term effects of clear‐cutting in mature secondary growth redwood forests prohibit their establishment. This may be due in part to the lower abundances of redwood‐specialist flowering plants such as redwood sorrel that we found in AM and NR forests (Table 1), as the abundance of critical floral resources directly affects bee abundance and biodiversity (Hegland & Boeke, 2006). The increase in rare bees found in OG forests may also be related to the availability of nesting substrate in this habitat. For example, we found a higher diversity of cavity‐nesting bees (*C. acantha* and *Osmia*) in OG forest than in NR or AM forest (Table A1: Appendix). Both *C. acantha* and *Osmia* species in other habitats are known to nest in pithy stems (Michener, 2007), and have been associated with elderberry trees (McIntosh, 1996), of which the blue elderberry (*Sambucus cerulea*) is associated with mature old‐growth redwood forest but not AM or NR forests (Russell & Michels, 2011). Some cavity‐nesting bees also use woody debris or deadwood as nesting substrate, however our habitat with the highest amount of large woody debris (LWD) was the NR forest type (Table 1), which had a lower species richness of cavity‐nesting bees than did the OG forest type (Table A1: Appendix), though higher than AM forests. This contrasts with other studies in coniferous forests showing that increases in coarse woody debris are associated with higher biodiversity of cavity‐nesting bees (Gelles et al., 2022). We also found a higher species richness of bees that are known to nest in the ground in other habitats (Michener, 2007) in OG forests, like *Lasioglossum* and *Halictus tripartitus* (Table A1: Appendix). It is well established that the abundance and diversity of ground‐nesting bee species increases with the cover of bare ground (Decker & Harmon‐Threatt, 2019; Felderhoff et al., 2023; Quistberg et al., 2016). Bare ground was very rare in the mature redwood forests that we studied, as the forest floor was frequently covered with a layer of fallen redwood needles, and denser secondary coniferous forests have a thicker layer of leaf litter (Willett, 2001). It is possible that OG forests provide more bare ground nesting substrate than AM or NR forests along creeks and river banks running through the forest floor, as OG redwood forests generally have more stable banks than in NR or AM forest types (Benda et al., 2002). Future studies should investigate the nesting substrate preferences of bees in mature redwood forests, and compare the availability of these nesting substrates in OG, NR, and AM forest types.

Overall, bipartite bee‐plant interaction networks in redwood forests are characterized by small size and low complexity (Figure 5; Table 4), which contrasts with networks from more open, meadow‐like ecosystems (Bascompte et al., 2003; Olesen et al., 2007). Due to the higher species richness of bees in OG forest plots (Figure 3), we expected that OG networks would exhibit higher nestedness and specialization than random networks (Olito & Fox, 2015). Studies in temperate evergreen broad‐leaved forests in Chile found significantly nested pollination networks, which contrasts with our findings in redwood forests (Ramos‐Jiliberto et al., 2009). Contrary to our expectations, nestedness and specialization were significantly lower than expected in both OG and NR forests (Table 4). This may be due to the generality of redwood sorrel, which was visited by almost all the bee species in the OG network. The generality of redwood sorrel could have reduced nestedness and specialization indices in OG forests. The long‐term effects of historical clear‐cutting and habitat fragmentation of the once widespread redwood forest may be reducing network complexity in mature old‐growth redwood forest bee‐plant interaction networks. In a study of a temperate coniferous forest/cropland matrix landscape, Gómez‐Martínez et al. (2020) found that nestedness and specialization of pollination networks was reduced as habitat fragmentation increased, and generalist species became more dominant. Studies in tropical forests have also found that network specialization of plant‐pollinator networks decreases in highly fragmented forests (Ferreira et al., 2020). Nestedness in other ecosystems also decreases with increased habitat loss and fragmentation (Traveset et al., 2018). Robustness was also significantly lower than expected in OG forest plots (Table 4). Many plants in the OG forest network were only visited by one bee species (though the identity of that bee species could vary between plants), and thus simulated removal of a single pollinator species could result in the theoretical removal of the dependent plant from the network, leading to lower robustness than expected. A strong exception to this was redwood sorrel, which was visited by many bee species and is thus more robust to removal of pollinators from the system (Figure 5).

Interestingly, the centrality of some bee species changed depending on the forest type (Figure 6). The lower normalized degree of *Andrena* sp. in OG forests as compared to other forest types may indicate a preference for redwood sorrel when it is present in significantly higher abundances, as in OG forest (Table 1). In OG forests, *Lasioglossum* sp. A emerges as an important generalist species as it interacts with 50% of all possible partners in the OG network (Figure 6). It is of note that this species was not found at all in AM forests, suggesting that AM forests may benefit from efforts to increase the abundance of this generalist species (González et al., 2010). The loss of “connector” species may lead to fragmentation of a pollination network into smaller networks that are more vulnerable to disturbance (González et al., 2010). Thus, monitoring and/or targeted restoration for connector species like *Bombus melanopygus*, and *Andrena* sp., both ground‐nesting species, may be worthwhile for land managers of redwood forests. While *Nomada* sp., also emerged as an important connector in NR forests, it likely parasitizes *Andrena* sp., and thus any efforts to support *Andrena* sp. would likely also support *Nomada* sp. The relationship between *Andrena* sp. and *Nomada* sp. in redwood forests is of note and merits further study, as parasite–host dynamics can have important consequences for rare pollinators by limiting the population of abundant species (Brown, 2022) and kleptoparasitic bees may respond negatively to anthropogenic disturbance (Graf et al., 2022).

Our results suggests that there are potential long‐term negative effects of historic clear‐cutting on bee biodiversity in mature secondary‐growth redwood forests. Forest management practices might benefit from mitigating these long‐term effects by enhancing native plant species composition to provide food for native bees, for example by planting high centrality plant species such as redwood sorrel or protecting existing flower patches from trampling in public use areas with signage. The species‐level centrality indices of plants and pollinators in bipartite plant‐pollinator interaction networks can in some cases be used to inform targeted conservation efforts, as high centrality pollinators may contribute to the overall ecosystem‐level stability of pollination services (Crespo et al., 2022; Elle et al., 2012; González et al., 2010; Ramos‐Jiliberto et al., 2009). High‐centrality and highly abundant bee species in OG forests, such as *Lasioglossum* sp. A (Table 2; Figure 6), may be important for the stability of pollination services in mature redwood forests, and thus warrant further study into their taxonomy and natural history in mature redwood forest. Future studies should also seek to learn more about the abundance and distribution of bees and nesting substrate in mature redwood forests, investigate bees in different seral stages of secondary forest following timber extraction activities, and test methods for habitat restoration targeting floral resources and nesting substrate for native bees in redwood forests. Targeted restoration to improve floral resource abundance and nesting substrate availability for bees in coniferous forests has been shown to improve bee abundance (Eckerter et al., 2021), and this study provides the necessary groundwork for future research into viable restoration methods for bees and pollination services in redwood forests.

## AUTHOR CONTRIBUTIONS

**Nya Ealy:** Data curation (equal); formal analysis (lead); investigation (lead); methodology (equal); project administration (supporting); supervision (equal); validation (equal); visualization (equal); writing – original draft (equal); writing – review and editing (equal). **Jaime Pawelek:** Data curation (equal); formal analysis (equal); investigation (equal); methodology (equal); validation (equal); visualization (equal); writing – original draft (equal); writing – review and editing (equal). **Jenny Hazlehurst:** Conceptualization (lead); data curation (equal); formal analysis (equal); funding acquisition (lead); investigation (equal); methodology (lead); project administration (equal); resources (equal); supervision (equal); validation (equal); visualization (equal); writing – original draft (equal); writing – review and editing (equal).

## CONFLICT OF INTEREST STATEMENT

The authors have no conflicts of interest to declare.

## Data Availability

All data files are openly available on Dryad at https://doi.org/10.5061/dryad.73n5tb32f.

## Appendix

**TABLE A1:** Number of native bee specimens captured per species and sex across all bee sampling plots and coast redwood forest types (actively managed secondary growth forest, naturally regenerating secondary growth forest, and old growth forest) in the Santa Cruz Mountains of California.

Family	Species	Female	Male	Worker
Apidae	*Bombus melanopygus*	0	1	5
	*Bombus vosnesenskii*	0	0	1
	*Ceratina acantha*	6	0	
	*Nomada* sp.	11	12	
Halictidae	*Halictus tripartitus*	1	0	
	*Lasioglossum* (*Evylaeus*) sp. A	29	0	
	*Lasioglossum* (*Evylaeus*) sp. B	1	0	
	*Lasioglossum* (*Evylaeus*) sp. E	1	0	
Megachilidae	*Osmia* (*Melanosmia*) sp.	0	8	
	*Osmia* (*Melanosmia*) sp. A	2	0	
	*Osmia* (*Melanosmia*) sp. C	1	0	
Andrenidae	*Andrena* sp.	18	9	
	Total	70	30	6

*Note*: The European honey bee (*Apis mellifera*) was observed at all study sites, but was not recorded.

**TABLE A2:** Key characters used to differentiate the six morphospecies of two genera captured in bee sampling plots through sweep‐netting.

Genus	Morph	Key characters
Lasioglossum (*Evylaeus*)	sp. A	Body length 6–7 mm; long face; dense pitting on scutum; propdeum with longitudinal regulae nearly reaching apical edge of segment; no hair banding on abdomen
*Lasioglossum* (*Evylaeus*)	sp. B	Body length ~ 5 mm; dense pitting on scutum; propodeum rounded over with short longitudinal striations covering basal 1/3 to 1/2 of segment
*Lasioglossum* (*Evylaeus*)	sp. E	Body length ~ 5 mm; face round; dense pitting on scutum; propodeum squared off on apical lateral corners with basal longitudinal striations, T2 with pale hair patches on basal lateral sides, stigma brown
*Osmia* (*Melanosmia*)	sp. A	Body length ~ 8 mm; metallic blue color; mixture of pale and black hairs on head and thorax; thin apical band without punctures on apical edge of terga; scopa black; hairs on pleura pale
*Osmia* (*Melanosmia*)	sp. B	Body length 8–9 mm; hair on head nearly all black, with a few pale hairs around antennal bases; hairs on thorax mostly pale, with some black hairs mixed in; T1 covered in pale hairs, remaining terga with pale, sparse hairs on apical edge and black hairs covering disk; wide impunctate, apical bands, tessellate; black scopa; hairs on pleura black
*Osmia* (*Melanosmia*)	sp. C	Body length 8–9 mm; hair on face black, hair on thorax mostly black with patch of pale hairs under wing attachment; pleura black, long, pale hairs on T1; narrow, tessellate impunctate band on terga; scopa black

*Note*: Shown are the genus of the morphospecies (Genus), the unique name given to each morphospecies (Morph), and the key characters used to differentiate that morphospecies from other morphospecies of the same genus (Key Characters).

**TABLE A3:** Bee species by sex (Female, Male, or Worker) captured in “bee bowls” or pan traps placed at 10 different randomly selected locations per sampling trip per forest over three sampling trips per year from 2021 to 2022 in three forest types.

Forest type	Bee species	Female	Male	Worker
AM (*N* = 60)	*Andrena* sp.	1	0	0
	*Nomada* sp.	1	2	0
	*Osmia* sp.	0	1	0
NR (*N* = 60)	*Osmia* sp.	0	1	0
OG (*N* = 60)	*Andrena* sp.	2	2	0
	*Bombus vosnesenskii*	0	0	1
	*Lasioglossum* sp. B	1	0	0
	*Osmia* sp.	0	1	0

*Note*: Each pan trap station consisted of one bowl each of yellow, blue, and white color filled halfway with soapy water. Pan traps were left out from 11:00 am until 6:00 pm at each session to encompass the warmest part of the day. Pan trap capture rates were extremely low, and thus data was not analyzed.

Abbreviations: AM, mature secondary growth redwood forest with active management through retention forestry; NR, mature secondary growth redwood forest, naturally regenerating; OG, mature old growth redwood forest.

**TABLE A4:** Results of coverage‐based estimation Hill number estimation for 95% coverage level for species richness, shannon diversity, and simpson diversity indices in different redwood forest types.

Forest type	Index	Observed	Estimator	SE	LCL	UCL
AM	Species richness	3.00	3.44	1.20	3.03	10.66
AM	Shannon diversity	2.34	2.75	0.89	2.34	4.49
AM	Simpson diversity	1.98	2.25	0.89	1.98	4.00
NR	Species richness	5.00	5.00	0.50	5.00	6.49
NR	Shannon diversity	4.15	4.46	0.40	4.15	5.25
NR	Simpson diversity	3.77	4.19	0.53	3.77	5.22
OG	Species richness	10.00	14.43	7.09	10.49	50.34
OG	Shannon diversity	6.25	6.95	0.89	6.25	8.69
OG	Simpson diversity	4.75	5.04	0.73	4.75	6.46

*Note*: Observed refers to the observed value of the index, and Estimator refers to the estimated value of the index at the 95% coverage level.

Abbreviations: AM, actively managed; LCL, lower confidence level; NR, naturally regenerating; OG, old growth forest; SE, standard error; UCL, upper confidence level.
